# From Hemolysis to Lupus: A Case of Evans Syndrome Revealing Systemic Autoimmunity

**DOI:** 10.1002/ccr3.72217

**Published:** 2026-03-06

**Authors:** Biruk T. Mengistie, Chernet T. Mengistie, Mikiyas G. Teferi, Daniel C. Teka, Alefe Kedame Godifey, Abel G. Wubie, Bruke Z. Abenet

**Affiliations:** ^1^ School of Medicine College of Health Sciences, Addis Ababa University Addis Ababa Ethiopia; ^2^ School of Medicine College of Health Sciences, Mekelle University Mekelle Ethiopia; ^3^ College of Medicine and Health Sciences Bahir Dar University Bahir Dar Ethiopia; ^4^ Department of Family Medicine Atrium Health, Navicent Macon Georgia

**Keywords:** autoimmune cytopenia, Evans syndrome, hemolytic anemia, systemic lupus erythematosus, thrombocytopenia

## Abstract

Evans syndrome (ES), the coexistence of autoimmune hemolytic anemia and immune thrombocytopenia, can unmask systemic autoimmune disease. We report a 30‐year‐old woman who presented with fatigue, jaundice, pallor, and mucocutaneous bleeding. Laboratory evaluation demonstrated severe Coombs‐positive hemolytic anemia (hemoglobin 5.8 g/dL, reticulocytosis, high LDH, undetectable haptoglobin) and marked thrombocytopenia, whereas immunologic testing revealed high‐titer ANA, anti‐dsDNA positivity, and low complement levels; infections, thrombotic microangiopathy, and malignancy were excluded. She was stabilized with transfusion and treated with high‐dose corticosteroids and intravenous immunoglobulin, achieving a partial response. Persistent cytopenias prompted rituximab, after which blood counts normalized and hemolysis resolved without major infectious or thrombotic events. At 6 months, she remained in remission on maintenance therapy. This case emphasizes that ES can be the initial manifestation of systemic lupus erythematosus and supports early autoimmune evaluation and tailored immunosuppression to address both cytopenias and underlying disease.

## Key Clinical Message

Evans syndrome can be the presenting feature of systemic lupus erythematosus. Prompt recognition of Coombs‐positive hemolytic anemia with immune thrombocytopenia, exclusion of alternative causes (infection, malignancy, and TTP, which can be Coombs‐negative), and early immunosuppression tailored to both the cytopenias and underlying autoimmunity, with rituximab for refractory cases, improve outcomes and warrant long‐term follow‐up.

## 1 Introduction

Evans syndrome (ES) is defined by the simultaneous or sequential occurrence of at least two autoimmune cytopenias, typically warm autoimmune hemolytic anemia (AIHA) and immune thrombocytopenia (ITP) [1, 2]. It is an uncommon, often chronic autoimmune disorder with a relapsing course [1, 3]. Current estimates place the incidence of ES at roughly 1–2 per million person‐years, with a prevalence on the order of tens of cases per million [4]. For perspective, ES occurs at an incidence approximately 10–30 times lower than isolated ITP or AIHA [2, 4]. The syndrome can be primary (idiopathic) or secondary to other diseases [4, 5]. In children, ES often reflects inborn immunodeficiencies or autoimmune lymphoproliferative syndrome (ALPS) [1]. In adults, up to a quarter of ES cases are secondary to systemic disorders, most commonly autoimmune diseases (e.g., systemic lupus erythematosus [SES], common variable immunodeficiency, or hematologic malignancies) [4]. Indeed, SLE is a well‐recognized association: published series reports that 1.7%–2.7% of patients with SLE develop ES during their disease course [6].

Clinically, ES presents with features of hemolytic anemia (fatigue, pallor, and jaundice) and thrombocytopenia (mucosal bleeding and petechiae) [1, 7]. The direct antiglobulin test (Coombs test) is typically positive in AIHA, and markers of hemolysis (elevated LDH, indirect bilirubin, and low haptoglobin) are evident [8]. By definition, two cytopenias must be present (e.g., hemoglobin < 10 g/dL and platelets < 100 × 10^9^/L) and secondary causes ruled out. No formal diagnostic “criteria” exist beyond these findings [9]. A broad diagnostic evaluation is advised: recent expert consensus recommends bone marrow examination and imaging to exclude occult malignancy or marrow failure in new ES diagnoses [3, 8].

Therapeutically, ES is treated empirically with immunosuppression. First‐line therapy is high‐dose corticosteroids; intravenous immunoglobulin (IVIG) may be added, particularly if severe thrombocytopenia or bleeding is present [1, 3]. Because ES often relapses, steroid‐sparing agents (rituximab, mycophenolate, cyclosporine, or other immunosuppressants) are commonly employed as second‐line therapy [1, 3, 9]. In refractory cases, splenectomy or other interventions (e.g., thrombopoietin mimetics) may be considered, though no randomized trials guide management [3, 9].

In patients with ES, SLE should be strongly considered. Hematologic abnormalities are integrated into SLE classification criteria and can be the initial manifestation [5]. We report an adult patient whose ES revealed previously unrecognized SLE. This case illustrates the epidemiology, diagnostic nuances, and management of ES, and underscores the importance of evaluating for underlying autoimmune disease.

## 2 Clinical History/ Examination

A 43‐year‐old previously healthy woman presented with a 3‐week history of progressive dyspnea on exertion, profound fatigue, and yellowish discoloration of the eyes. She denied recent drug exposure, transfusions, or a family history of hematologic disease.

On physical examination, she appeared pale and mildly icteric. Her vital signs showed tachycardia with a pulse rate of 110 beats per minute, blood pressure of 100/65 mmHg, respiratory rate of 24 breaths per minute, and oxygen saturation of 94% on room air. Mucosal pallor was evident, and there were scattered petechial lesions over the lower extremities. A faint erythematous rash was noted over her malar area, which she reported had been recurrent for several months but had never been previously evaluated. Oral examination revealed shallow ulcers over the hard palate. Abdominal examination demonstrated splenomegaly, while the liver was not enlarged. Cardiovascular and respiratory examinations were otherwise unremarkable.

## 3 Differential Diagnosis, Investigations, and Treatment

Laboratory evaluation revealed severe normocytic anemia (hemoglobin 5.8 g/dL, hematocrit 18%) with marked reticulocytosis (24.9%), thrombocytopenia (74 × 10^9^/L), and leukocytosis (22.5 × 10^9^/L) with neutrophilia. Biochemical markers confirmed ongoing hemolysis, including indirect hyperbilirubinemia (total bilirubin 2.32 mg/dL, direct 0.80 mg/dL), elevated lactate dehydrogenase (820 U/L), and undetectable haptoglobin (0.03 g/L). Direct antiglobulin test (DAT) was strongly positive (IgG +4, C3d +2), and the indirect Coombs test was also positive (+3), supporting autoimmune hemolysis. Bone marrow aspiration showed reactive erythroid hyperplasia without evidence of malignancy. Infectious screening was negative for HIV, HCV, and active HBV; EBV IgG was reactive, consistent with prior exposure. Inflammatory markers were markedly raised (ESR 80 mm/h, CRP 60.5 mg/L). A summary of the patient's investigations is provided in Table 1.

**TABLE 1:** Admission laboratory investigations (selected).

Test	Reference range	Result
HCT (%)	37.0–51.0	18
Hb (g/dL)	12.0–14.0	5.8
RBC (×10^12^/L)	4.20–6.30	1.4
MCV (fL)	80.0–97.0	98.0
PLT (×10^9^/L)	140–440	74
WBC (×10^9^/L)	4–11	22.5
NEUT (×10^9^/L)	1.8–7.7	16.8
Reticulocytes (%)	—	24.9
Total bilirubin (mg/dL)	0–1.2	2.32
Direct bilirubin (mg/dL)	0–0.2	0.80
LDH (U/L)	135–214	820
Haptoglobin (g/L)	0.3–2.0	0.03
Direct Coombs (DAT)	—	IgG +4/C3d +2
Indirect Coombs (ICT)	—	+3
ESR (mm/h)	0–20	80
CRP (mg/L)	0–5	60.5
AST (U/L)	0–32	68
ALT (U/L)	10–33	28
ANA	—	Positive (high titer)
Anti‐dsDNA	—	Positive
Complement C3/C4	—	Low/Low

Abbreviations: HCT, hematocrit; Hb, hemoglobin; RBC, red blood cell count; MCV, mean corpuscular volume; PLT, platelet count; WBC, white blood cell count; NEUT, neutrophils; LDH, lactate dehydrogenase; DAT, direct antiglobulin test; ICT, indirect Coombs test; ESR, erythrocyte sedimentation rate; CRP, C‐reactive protein; ANA, antinuclear antibody.

The combination of autoimmune hemolytic anemia and immune thrombocytopenia established the diagnosis of ES, after exclusion of secondary causes, such as infections, lymphoproliferative disorders, and drug‐induced cytopenias. Concurrent clinical features (malar rash, oral ulcers, and splenomegaly) and immunological findings confirmed SES: ANA was positive at a high‐titer, anti‐double stranded DNA antibody was positive and complement levels (C3 and C4) were markedly reduced.

The patient was admitted and stabilized with red blood cell transfusions. She was started on high‐dose intravenous corticosteroids, followed by a tapering oral regimen and hydroxychloroquine. Supportive therapy included folate supplementation and gastric protection.

## 4 Outcome and Follow‐Up

Over the hospital course, her jaundice resolved, her hemoglobin gradually improved, and platelet counts increased. She was discharged clinically stable on oral steroids and hydroxychloroquine, with close outpatient follow‐up by hematology and rheumatology. At two‐month follow‐up, she remained stable with no recurrence of severe cytopenias and continued monitoring for potential SLE organ involvement.

## 5 Discussion

Evans syndrome as the initial presentation of SES is rare [9]. In large cohorts, only a small fraction of patients with SLE develop ES. For example, a Chinese case–control study of 5724 hospitalized patients with SLE found ES in only 27 individuals (0.47%) [10]. In that series, ES appeared at SLE diagnosis in roughly 30% of affected patients, whereas others developed ES later. These figures align with reports that approximately 1%–3% of patients with SLE experience ES during their disease course [1, 6, 10]. Thus, initial SLE manifesting as ES is uncommon but documented. Awareness is crucial since early hematologic involvement can precede or obscure other lupus features [11].

Diagnosing ES in the context of possible SLE poses challenges. Clinically, ES must be distinguished from other causes of cytopenias (e.g., thrombotic microangiopathies, infections, and nutritional deficiencies) [3, 9]. Expert guidance recommends an extensive workup: in addition to hematologic evaluation, bone marrow biopsy and imaging (e.g., CT) are advised to exclude marrow malignancy or splenic pathology [3, 8]. In suspected SLE, detailed immunologic testing is essential. Clues favoring SLE‐associated ES include a positive ANA, anti‐dsDNA or other lupus‐specific antibodies, and complement consumption [9, 12]. Indeed, in published cases of SLE‐associated ES, hypocomplementemia and elevated serum IgG were notably common [10]. In our patient, detection of ANA and anti‐dsDNA antibodies with low C4 confirmed underlying SLE. The “hematologic” criterion for SLE (Coombs‐positive hemolysis and thrombocytopenia) is well established, so evaluation for lupus is mandatory when ES is diagnosed [12].

Therapeutically, ES often responds to standard immunosuppression, though evidence is limited [9]. Corticosteroids induce remission in the majority of patients initially. In one SLE cohort, 92.6% of ES cases improved on prednisone plus additional immunosuppressive agents [10]. IVIG is frequently added when bleeding or refractory thrombocytopenia occurs [1]. Despite initial responses, many patients eventually experience relapse, making it necessary to escalate treatment beyond corticosteroids to second‐line agents [7]. Rituximab (anti‐CD20) has emerged as a key agent: expert consensus strongly recommends rituximab for refractory warm AIHA and ITP in ES [13]. In SLE‐associated ES specifically, rituximab leads to complete or partial responses in many cases, though relapses occur [3, 7]. In a multicenter study of 71 patients with SLE with immune cytopenias, 60% of those with ES achieved an initial response to rituximab [7]. Durability was limited, with nearly 40% relapsing over follow‐up, but retreatment was often effective. Other immunosuppressants (azathioprine, mycophenolate, cyclosporine, etc.) have been used in refractory cases by analogy with SLE management [1, 3]. Splenectomy and thrombopoietin receptor agonists are less commonly employed now, given infection and thrombosis risks [14, 15].

Prognostically, ES carries a guarded outlook, worse than isolated ITP or AIHA [3, 4]. In the Danish cohort (1977–2017), median survival after ES diagnosis was only 7.2 years; notably, secondary ES (reflecting underlying disease) had a median survival of 1.7 years (5‐year survival ~38%), far worse than primary ES (median ~ 10.9 years) [4]. Thus, secondary ES (as in SLE) tends to have higher mortality, often due to bleeding, infection or complications of therapy [4, 9]. In contrast, SLE‐associated ES may fare better than idiopathic ES, likely because concurrent SLE therapies (antimalarials and immunosuppressants) also mitigate ES recurrences [6, 16]. In our case, the timely institution of aggressive immunotherapy led to durable remission. Nonetheless, vigilance is required: relapse of ES or SLE flares may occur, and patients warrant long‐term follow‐up.

Overall, this case adds to a growing literature on ES in lupus. It underscores that although rare, ES can be the presenting sign of SLE. Differentiating primary ES from SLE‐related ES relies on careful evaluation for systemic features and serologies [6, 8]. Management should address both the cytopenias and the underlying autoimmune disease [9, 13]. Early use of corticosteroids and adjunctive therapies (IVIG, rituximab) can achieve remission, but close follow‐up is key given the risk of relapse [7, 13].

## 6 Conclusion

This case emphasizes that ES, though rare, may be the initial manifestation of SES in adults. Recognition of ES should prompt a thorough search for underlying autoimmunity, including lupus‐specific serology and complement levels, as hematologic criteria are integral to SLE diagnosis. The diagnostic workup must also exclude other causes of pancytopenia (e.g., marrow malignancy or thrombotic microangiopathy). Treatment requires aggressive immunosuppression: corticosteroids (often combined with IVIG) should be started promptly, and therapy escalated (e.g., rituximab or second‐line agents) if response is inadequate. Despite therapy, ES carries a risk of relapse and complications, underscoring the need for vigilant long‐term follow‐up. Clinicians should maintain a high index of suspicion for SLE in any patient presenting with ES, as early diagnosis of SLE can guide additional therapy and improve outcomes.

## Author Contributions

**Biruk T. Mengistie:** conceptualization, data curation, writing – original draft. **Chernet T. Mengistie:** conceptualization, writing – original draft. **Mikiyas G. Teferi:** visualization, writing – review and editing. **Daniel C. Teka:** data curation, resources. **Alefe Kedame Godifey:** writing – original draft, writing – review and editing. **Abel G. Wubie:** data curation, writing – review and editing. **Bruke Z. Abenet:** supervision.

## Funding

The authors have nothing to report.

## Ethics Statement

IRB review and approval were waived for this case report.

## Consent

Written informed consent was obtained from the patient for publication of this case report and any accompanying images. A copy of the written consent is available for review by the editor‐in‐chief of this journal.

## Conflicts of Interest

The authors declare no conflicts of interest.

## Data Availability

The data underlying the results presented in this work are available within the manuscript.

## References

[1] J. C. Jaime‐Pérez , P. E. Aguilar‐Calderón , L. Salazar‐Cavazos , and D. Gómez‐Almaguer , “Evans Syndrome: Clinical Perspectives, Biological Insights and Treatment Modalities,” Journal of Blood Medicine 9 (2018): 171–184. 30349415 10.2147/JBM.S176144 PMC6190623

[2] D. Jiang and D. J. Kuter , “Evans syndrome revisited,” Blood Reviews 74 (2025): 101322. 40717001 10.1016/j.blre.2025.101322

[3] B. Fattizzo , M. Marchetti , M. Michel , et al., “Diagnosis and Management of Evans Syndrome in Adults: First Consensus Recommendations,” Lancet Haematology 11, no. 8 (2024): e617–e628. 38968944 10.1016/S2352-3026(24)00144-3

[4] D. L. Hansen , S. Möller , K. Andersen , D. Gaist , and H. Frederiksen , “Evans Syndrome in Adults ‐ Incidence, Prevalence, and Survival in a Nationwide Cohort,” American Journal of Hematology 94, no. 10 (2019): 1081–1090. 31292991 10.1002/ajh.25574

[5] M. Mansour , A. Shamasnah , D. Alsaadi , S. Abu Saif , and A. Krama , “Evans Syndrome as a Presentation in Systemic Lupus Erythematous, Coexisting With Hashimoto's Thyroiditis and Pernicious Anemia: A Case Report,” Journal of Medical Case Reports 18, no. 1 (2024): 643. 39732732 10.1186/s13256-024-05002-3 PMC11682623

[6] G. L. Costallat , S. Appenzeller , and L. T. L. Costallat , “Evans Syndrome and Systemic Lupus Erythematosus: Clinical Presentation and Outcome,” Joint, Bone, Spine 79, no. 4 (2012): 362–364. 21944976 10.1016/j.jbspin.2011.07.004

[7] M. Michel , V. Chanet , A. Dechartres , et al., “The Spectrum of Evans Syndrome in Adults: New Insight Into the Disease Based on the Analysis of 68 Cases,” Blood 114, no. 15 (2009): 3167–3172. 19638626 10.1182/blood-2009-04-215368

[8] U. Jäger , W. Barcellini , C. M. Broome , et al., “Diagnosis and Treatment of Autoimmune Hemolytic Anemia in Adults: Recommendations From the First International Consensus Meeting,” Blood Reviews 41 (2020): 100648. 31839434 10.1016/j.blre.2019.100648

[9] S. Audia , N. Grienay , M. Mounier , M. Michel , and B. Bonnotte , “Evans' Syndrome: From Diagnosis to Treatment,” Journal of Clinical Medicine 9, no. 12 (2020): 3851. 33260979 10.3390/jcm9123851 PMC7759819

[10] L. Zhang , X. Wu , L. Wang , et al., “Clinical Features of Systemic Lupus Erythematosus Patients Complicated With Evans Syndrome: A Case‐Control, Single Center Study,” Medicine (Baltimore) 95, no. 15 (2016): e3279. 27082565 10.1097/MD.0000000000003279 PMC4839809

[11] J. C. Santacruz , M. J. Mantilla , I. Rueda , S. Pulido , G. Rodriguez‐Salas , and J. Londono , “A Practical Perspective of the Hematologic Manifestations of Systemic Lupus Erythematosus,” Cureus 14, no. 3 (2022): e22938. 35399432 10.7759/cureus.22938 PMC8986464

[12] M. Aringer , K. Costenbader , D. Daikh , et al., “2019 European League Against Rheumatism/American College of Rheumatology Classification Criteria for Systemic Lupus Erythematosus,” Arthritis & Rheumatology (Hoboken, N.J.) 71, no. 9 (2019): 1400–1412. 31385462 10.1002/art.40930 PMC6827566

[13] A. Serris , Z. Amoura , F. Canouï‐Poitrine , et al., “Efficacy and Safety of Rituximab for Systemic Lupus Erythematosus‐Associated Immune Cytopenias: A Multicenter Retrospective Cohort Study of 71 Adults,” American Journal of Hematology 93, no. 3 (2018): 424–429. 29247540 10.1002/ajh.24999

[14] N. Shen , J. Qiao , Y. Jiang , et al., “Thrombopoietin Receptor Agonists Use and Risk of Thrombotic Events in Patients With Immune Thrombocytopenic Purpura: A Systematic Review and Meta‐Analysis of Randomized Controlled Trials,” Biomedical Reports 20, no. 3 (2024): 44. 38357229 10.3892/br.2024.1732 PMC10865300

[15] F. Tahir , J. Ahmed , and F. Malik , “Post‐Splenectomy Sepsis: A Review of the Literature,” Cureus 12, no. 2 (2020): e6898. 32195065 10.7759/cureus.6898 PMC7059871

[16] B. Rivalta , D. Zama , G. Pancaldi , et al., “Evans Syndrome in Childhood: Long Term Follow‐Up and the Evolution in Primary Immunodeficiency or Rheumatological Disease,” Frontiers in Pediatrics 7 (2019): 304. 31396497 10.3389/fped.2019.00304 PMC6664023

